# Fecal Microbiota Transplantation in Liver Cirrhosis

**DOI:** 10.3390/biomedicines11112930

**Published:** 2023-10-30

**Authors:** Adrian Boicean, Victoria Birlutiu, Cristian Ichim, Olga Brusnic, Danusia Maria Onișor

**Affiliations:** 1 County Clinical Emergency Hospital of Sibiu, 550245 Sibiu, Romania; adrian.boicean@ulbsibiu.ro (A.B.); victoria.birlutiu@ulbsibiu.ro (V.B.); 2 Faculty of Medicine, Lucian Blaga University of Sibiu, 550169 Sibiu, Romania; 3 Department of Gastroenterology, University of Medicine, Pharmacy, Science, and Technology of Targu Mures, 540142 Târgu Mures, Romania

**Keywords:** fecal microbiota transplantation, microbiota, liver cirrhosis, microbiota transfer

## Abstract

The human gastrointestinal tract houses a diverse array of probiotic and pathogenic bacteria and any alterations in this microbial composition can exert a significant influence on an individual’s well-being. It is well-established that imbalances in the gut microbiota play a pivotal role in the development of liver diseases. In light of this, a new adjuvant therapy for liver diseases could be regulating the intestinal microbiota. Through fecal microbiota transplantation, patients whose microbiomes are compromised are treated with stool from healthy donors in an attempt to restore a normal microbiome and alleviate their symptoms. A review of cross-sectional studies and case reports suggests that fecal microbiota transplants may offer effective treatment for chronic liver diseases. Adding to the potential of this emerging therapy, recent research has indicated that fecal microbiota transplantation holds promise as a therapeutic approach specifically for liver cirrhosis. By introducing a diverse range of beneficial microorganisms into the gut, this innovative treatment aims to address the microbial imbalances often observed in cirrhotic patients. While further validation is still required, these preliminary findings highlight the potential impact of fecal microbiota transplantation as a novel and targeted method for managing liver cirrhosis. We aimed to summarize the current state of understanding regarding this procedure, as a new therapeutic method for liver cirrhosis, as well as to explain its clinical application and future potential.

## 1. Introduction

Mortality due to chronic liver diseases has increased exponentially to a recent global level of 2 million deaths anually [1]. The most affected are men, with 2 out of 3 deaths recorded among them. Liver diseases carry greater significance based on the substantial financial burden they impose on society, resulting in an annual expenditure exceeding USD 32 billion in the United States [2].

The imbalance of the gut microbiota may be exacerbated by liver diseases, which are mutually harmful to each other [3]. Due to the portal vein connecting the liver directly to the intestines, bacteria and their metabolites can affect the liver in healthy as well as diseased states [4]. Moreover, by releasing bile acids into the biliary system, the liver communicates with the intestine bidirectionally [5].

A number of novel approaches for modulating the microbiota have been proposed in recent years, having many promising outcomes in terms of efficacy and clinical application, including personalized diets, antibiotics, probiotics and prebiotics, stomach operations, phage therapy and fecal microbiota transplantation (FMT) [6,7,8,9,10,11].

In this study, FMT is investigated as a potential future treatment for one of the most well-known chronic liver diseases represented by liver cirrhosis (LC), together with LC-related complications like hepatic encephalopathy (HE).

Treatment for liver cirrhosis can vary depending on the underlying cause and the severity of the disease. At the moment, liver cirrhosis is considered the final stage in the progression of liver disease and modern treatments are not curative and have not significantly improved liver function. A first step is lifestyle modifications. Changes can slow disease progression and improve the quality of life but may not directly impact survival rates and may not be sufficient for advanced cirrhosis cases [12]. The effectiveness of medications in treating liver cirrhosis varies depending on the cause and the stage of the disease. Additionally, it is crucial to emphasize that the disease prognosis must never be underestimated, as it stands as a paramount factor requiring constant attention. Research has demonstrated that nanoindentation techniques are capable of assessing and tracking the nuclear deformations in hepatic cells, enabling the differentiation between samples from a healthy population and those afflicted with liver cirrhosis disorders [13,14]. Antiviral medications for viral hepatitis can slow disease progression [15], but medications still do not yield spectacular results. Liver transplantation remains the most effective treatment for advanced cirrhosis, with high survival rates. Although it is a life-saving treatment, it remains difficult to access for patients and is only performed in exceptional cases, with extremely long waiting lists. In cases where patients do receive this treatment, the chances of survival increase significantly, with one study even showing that those who underwent a liver transplant while in end-stage liver disease had a survival advantage of up to 13–17 years compared to those who did not have access to a transplant [16].

Due to the previously mentioned factors, now more than ever, there is a need to find solutions for the treatment of liver cirrhosis, and FMT is considered as a possible option. Although LC is not an official contraindication to FMT, in medical literature there are currently only a few reports of patients with LC who have undergone FMT, those reports being only listed without detailed descriptions [17,18].

As well as offering a new outlook on the potential of using FMT in hepatic cirrhosis more often in the future, the present review aims to summarize the latest evidence and data regarding the indications and methods of FMT in LC.

## 2. Short History of Fecal Microbiota Transplantation (FMT)

Inflammatory bowel disorders (IBD) with their most well-known forms represented by Crohn’s disease and ulcerative colitis, along with dysbiotic conditions like CDI, have been shown to respond well to FMT [19,20,21,22,23].

It is important to mention that this procedure started to be used hundreds of years ago. In the 4th century, according to Dr. Ge Hong’s book entitled *Zhou Hou Bei Ji Fang*, drinking fermented fecal matter or fecal water was prescribed to treat food poisoning, diarrhea and fever. In 1580, a Chinese doctor named Li Shizhen wrote a book entitled *Ben Cao Gang Mu*, in which he described the effective treatment of more than 20 conditions using fermented feces or fecal water [24]. It was Eiseman et al. who in 1958 reported that fecal water enema was effective in treating pseudomenbraneous colitis for the first time in the English literature [24].

Initially, fecal microbiota were prepared from fresh feces and administered directly to the gastrointestinal tract under either endoscopic or non-endoscopic guidance [25,26,27]. In recent years, cryopreserved preparations have largely replaced these forms of FMT, allowing the treatment to be introduced into mainstream medicine [25]. Recently, a variety of administration methods have been used, such as colonoscopy and endoscopy, as well as enema and capsule therapy [26].

## 3. The Gut–Liver Axis

The human gastrointestinal tract is littered with microorganisms and there is growing recognition that these microorganisms play a significant role in healthy and disease states. Thanks to the symbiotic relation between the gastrointestinal (GI) tract and its microbiota, the latter helps with digestion, vitamin production and resistance to pathogens, but also contains bacteria that may cause disease [28]. As we age, the gut microbiota changes dramatically in composition due to a wide array of environmental factors [29]. In the elderly, a decrease in *Bifidobacterium* and the *Bacteroides* group has been observed, which can implicitly be associated with a decrease in the degree of protection, with an increased risk of the predominance of certain unwanted types of bacteria [30]. Interestingly, a maintained stability of the microbiota appears to be associated with longevity [30].

Different pathologic conditions, including obesity, diabetes and colorectal cancer, have been linked to changes in intestinal microflora, this pattern being nowadays called dysbiosis [31,32,33]. The condition occurs when complex commensal communities are disturbed, resulting in insufficient immune priming [4]. *Escherichia coli* and *Streptococcus pneumoniae* can adhere excessively to the gut microbiome of the host when the sensitive commensal relation between the intestine microbiome and the host is disrupted [1,34]. In addition to the increasing intestinal permeability, microbiome imbalance can cause bacterial translocation, resulting in an increase of the immune response, which can negatively affect the integrity of the tight junction within the intestinal membrane [1,35].

Researchers have found that impaired gastrointestinal motility, systemic and local immunological dysfunction, portal hypertension as well as decreased gastric acid production and secretion, are primarily responsible or the derangement of gut microbiota that causes the increase of pathogenic bacterial populations and a decrease in commensal bacterial populations [36,37]. Numerous reports have found that patients with LC, autoimmune diseases, Parkinson’s disease, autism and *Clostridioides difficile* infections (CDI) have altered gut microbiotas [29,38,39]. Moreover, research studies have established the significant influence of gut microbiota in conditions such as anxiety, depression [40], multiple sclerosis [41], insomnia [42], polycystic ovarian syndrome [43] and diabetes [44]. This extensive involvement underscores the versatility of fecal microbiota transplantation (FMT), which offers approaches applicable to both intestinal and non-intestinal issues. FMT can be a valuable intervention for a wide range of metabolic, hormonal and neurodegenerative diseases.

**Figure 1:**
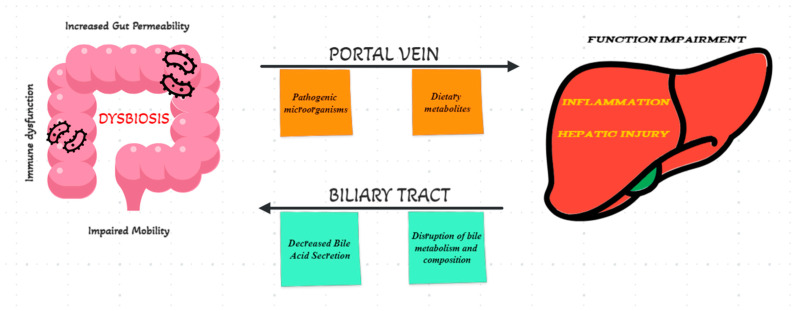
Schematic representation of the gut–liver axis.

The liver challenges bacteria from the gut and ingested foods as soon as they enter the bloodstream, as the first organ to do so [4]. The gut microflora has a profound effect on liver physiology and function and also plays a significant role in hepatic diseases, in their pathogenesis and progression. The portal circulation can be infiltrated by potentially pathogenic microorganisms and their products, thereby causing inflammation and hepatic injury [31]. On the other side, a person with LC may experience pathologic conditions such as decreased bile acid secretion, increased gut permeability, impaired motility and immune dysfunction, which may negatively affect intestinal microflora and lead to dysbiosis [31,45,46] (Figure 1). It seems that through signaling pathways (including the farnesoid X receptor and G protein-coupled membrane receptor 5), the gut microbiota exerts significant influence over both the composition of bile acids and the functioning of the liver [47]. Those with cirrhosis suffer from fundamentally altered gut microbiomes as a result of gut–liver axis dysfunction [48]. Moreover, by analyzing the feces of patients with hepatic cirrhosis, targeted changes in bacteria have been identified, with a reduction of the following species: *Ruminococcaceae* and *Lachnospiraceae* together with significant higher number in the levels of *Enterococcaceae*, *Enterobacteriaceae* and *Staphylococcaceae* [49].

## 4. Transplantation Techniques

In the medical community, there are still ongoing discussions regarding the ideal method for administering fecal microbiota transplantation. Various methods of transferring fecal matter have been described in detail, which can be used through both the upper gastrointestinal tract (e.g., naso–duodenal, naso–jejunal, etc.) and the lower gastrointestinal tract (enema or colonoscopy up to the cecum), but studies on their effectiveness are divided [50,51]. Gulati et al. emphasize that the choice of transfer method should be made based on multiple criteria, taking into account patient preferences, available methods, patient age and the severity of the condition. Their work also highlights the oral route of transplant administration, which is gaining ground due to studies showing promising results in terms of efficiency [52,53]. The FDA has already granted approval for the market entry of a product known as Vowst, which has therapeutic implications for modifying the microbiome in patients with CDI. The administration is oral and exceptionally straightforward and it could become a promising treatment for modulating the microbiota in individuals with liver cirrhosis in the future [54]. However, in many centers, colonoscopy remains the preferred method, especially in CDI, where the feces can be administered directly at the cecal level, thus traversing the entire colon [51]. Most clinical studies for FMT efficacy in conditions other than CDI tend to avoid using colonoscopy as the method of administration. One of the probable reasons is the lower patient compliance and their fear of pain, along with the awareness of the risks associated with this method noted by research physicians. These risks include less severe adverse reactions such as abdominal pain, intestinal transit disorders and flatulence, but also more severe adverse reactions such as perforation or transmission of highly aggressive pathogens [55,56].

Currently, considerable focus is placed on performing FMT using oral capsules. Undoubtedly, regardless of whether they access the upper or lower GI tract, this method carries much lower risks compared to invasive procedures [57,58]. However, the issue of preparing the transplant material arises, which varies depending on the route of administration and most importantly, the level of preparation that may alter the donor’s microbiota and lead to significantly reduced efficacy of the procedure.

Hamilton et al. propose that standardizing transplant methods brings practical benefits without eluding procedure effectiveness [59]. However, in order to draft high-quality protocols, future studies must compare the effectiveness of different transplantation methods and donor material preparations. Until then, transplantation of freshly prepared fecal matter dissolved in various solutions or fecal matter stored at −80 degrees Celsius remains the safest and most studied method for increased efficacy [60].

Choosing the donor remains a laborious process in many cases, due to hard-to-find volunteers and the multiple tests required to minimize risks [61,62]. In larger centers, the development of fecal microbiota banks has already begun, offering the advantage of quickly accessing these reserves in times of need and saving precious time if a suitable donor cannot be found for the patient [63].

Exclusion criteria for donors include recent antibiotic use within the last 30 days, and oncological, neurological, autoimmune and infectious pathologies. Donors should not be under 18 years of age, even though young donors have a high probability of compatibility and in most cases, do not present a medical history. Physicians must be constantly aware of the medico–legal implications for minor patients and closely follow the legislation in the country where they practice medicine [64].

## 5. Safety of FMT

Retrospective studies and case reports have shown the positive effect that FMT has on patients with LC. A limited number of safety data are available about this procedure in the long term. Fecal microbiota is prepared by following a standard methodology that incorporates a donor selection process that ensures the healthiest donors are selected and the donor material is rigorously tested in a laboratory prior to the microbiota preparation process [59,65]. Detailed medical certificates and medical histories, stool tests, serological tests and, most importantly, the donor’s informed consent, must be provided before surgery [27,65,66]. In Table 1, a summary of donor screening is provided.

Especially when initiating the transplant procedure for patients with severe liver impairment, careful selection is vital in every center. Risks associated with transplanting cirrhotic patients may differ from those with other pathologies [67]. It should be noted that there is insufficient literature documenting the specific interaction between fecal microbiota transplantation and liver cirrhosis. This aspect should be explained to patients to make them aware of the potential risks they are exposing themselves to [68]. Various degrees of liver impairment may respond differently to treatment and there are studies in the literature reporting deaths among patients with decompensated liver cirrhosis who underwent FMT [17,69]. Although it could be coincidental and the procedure might not have directly influenced the worsening of patients’ conditions leading to unfavorable outcomes, it is crucial to recognize that any additional, especially invasive, procedure might carry more risks than benefits. In the future, there is a need for a tailored risk set based on each pathology, as the differences are significant among them. Of course, it is essential not to forget that pathology must be interpreted in context. Information such as age, gender, body mass index, as well as other personal medical histories, should also be considered and neglecting them could be fatal for the patient.

## 6. Liver Cirrhosis (LC)

Chronic liver diseases can be attributed to viruses, alcohol abuse, obesity, autoimmune diseases, toxins, as well as genetics and environmental influences [70]. Meanwhile, any changes in the gut microbiome may adversely impact the liver’s homeostatic functions, leading to disorders of liver function and the development of additional liver diseases [4]. Fatigued liver, hepatitis, fibrosis and LC are all diseases that are classified as chronic liver diseases [71].

The cause of necroinflammation and fibrogenesis in LC can vary due to various mechanisms. During histological examination, it is evident that hepatic vascular architecture is dramatically altered due to the proliferation of nodules around dense fibrotic septa, followed by the extinction of the parenchyma and liver structures’ collapse [72]. Several complications of LC are associated with the intestinal microbiome but are represented also by jaundice, HE, ascites, variceal bleeding, spontaneous bacterial peritonitis and infections [26,73]. Out of these, HE is the one that causes the most frequent hospital admissions, placing a heavy burden not only on the patient, but also on the caregivers and healthcare systems [74]. In patients with cirrhosis, hospitalizations are more common and antibiotic use is more prevalent, increasing their likelihood of contracting CDI [74].

For identifying patients with similar rates of progression and survival of cirrhosis, compensation and decompensation can be classified as simple and reproducible methods [73]. It is cirrhosis, regardless of etiology, that is, the liver disease progression end stage, which can result later in hepatocellular carcinoma development [75].

## 7. Hepatic Encephalopathy (HE)

A well-known and also one of the most common complications of LC, HE has a cumulative incidence of more than 40% during the course of the disease, with more than 14% of cases occurring when the disease is diagnosed and 21% of cases occurring in patients with decompensated stages [48,76]. The combination of systemic inflammation, excessive amount of endotoxins and ammonia contributes to HE pathogenesis [28,77]. In chronic liver disease due to ammonia toxicity, metabolic failure and portosystemic shunting are believed to be responsible [78].

Pathogenic bacteria that are more likely to undergo numerical growth in the gut microbiota of patients with hepatic cirrhosis and HE are primarily *Enterobacteriaceae*, *Porphyromonadaceae*, *Veillonellaceae* and *Alcaligenaceae*. At the same time, patients have a numerical decrease of commensal bacteria, including *Lachonospiraceae*, *Ruminococcaceae* and *Blautia* [28,37,79,80].

A variety of neuropsychiatric problems could be caused by HE, from subclinical changes to comas [76]. HE is primarily caused by the gut–liver–brain axis malfunctioning, so the treatment usually involves changing the microbiota’s composition and function [75,76]. As a result of recurrent HE, patients with LC are at greater risk of hospital readmission despite standard of care. This may contribute to irreversible neurocognitive impairment [4,81,82]. For patients who are not responding to traditional treatments like lactulose or non-absorbable antibiotics such as rifaximin, other options may be necessary [76,83]. Previously, FMT capsules were found to be an alternative method of treatment for the improvement of cognitive function and to reduce hospitalizations in the case of persons suffering from HE who were already taking lactulose and rifaximin [19,82]. There was a correlation between cognitive improvements and donor microbiota transfer to mucosa and stool [82]. In patients with HE, when these are combined with the standard therapy, FMT can modulate the gut microbiota [37]. FMT can be used to reduce ammonia production by restoring intestinal barrier integrity, decreasing intestinal uptake of ammonia through the alteration of the intestinal microbiota and by increasing the function of the liver in order to decrease ammonia clearance [78].

## 8. Impact of FMT in LC

A study conducted in 2015 by M. Grąt et al. wanted to provide new information regarding the importance of dysbiosis in pre-liver transplantation candidates. During the study, 40 patients with LC who underwent FMT were analyzed. In cirrhotic patients with pre-liver transplantation gut dysbiosis, *Enterococcus* was the most influencial factor of pre-liver transplantation gut dysbiosis, even though *Bifidobacterium* and *Enterococcus* abundance are related [31].

To determine the safety of rationally derived stool donors for men with recurrent HE, a randomized open-label clinical trial was conducted in 2017. When FMT was performed on those with cirrhosis with recurrent HE, there was a reduction in hospitalizations, an improvement in cognition, and a reduction in dysbiosis [19].

Another study in 2017 made by Philips et al. analyzed 195 patients suffering from alcoholic liver disease. Among patients with severe alcoholic hepatitis, FMT for one week improved the severity of the disease and survival, according to the study [84]. The research also found that 6–12 months after the procedure, there was coexistence of donor and recipient species, a finding confirmed also by Li et al. in 2016 [84,85].

Hospitalizations, prophylactic antibiotics and comorbidities in cirrhotic patients increase their risk of CDI [86]. The rising antibiotic resistance and the irrational use of antibiotics pose significant global challenges; therefore, the selection and utilization of these drugs must be well-founded [87]. Pringle et al. conducted a prospective study from 2013 to 2017 of patients receiving frozen FMT capsules for recurrent CDI with advanced stages of LC. The researchers found that to cure the recurrent CDI, there was a need of more doses of frozen oral FMT in cirrhotic patients [88].

In a 2018 retrospective case series by Mehta et al., they treated recurrent open HE with a single FMT treatment. Using fecal material from rigorous, patient-identified donors, 10 patients aged 25–65 years received a single FMT via colonoscopy. At week 20 post-treatment, six patients showed a sustained clinical response after receiving a single FMT treatment [69].

There were four cases of LC with CDI reported by Olmedo et al. in 2019, three of them having a FMT procedure via colonoscopy and one through a nasojejunal tube. After the procedure, two patients suffered serious complications, with one of them sadly dying as a result. Analyzing the cases in greater detail, it seems that the cholangitis that appeared later in the death case was merely a coincidence. In all three cases that survived FMT, during continuous follow-up periods of one, five and eleven months no recurrent episodes of CDI occurred [17].

A retrospective study in 2020 made by Cheng et al. analyzed 63 adults with LC who underwent FMT through colonoscopy for CDI between 2012 and 2018. The authors collected demographic information on patients and studied their characteristics related to LC, CDI and FMT and compared patient success rates at 8 weeks between patients with different stages of LC [89]. Previously, meta-analyses and systematic reviews of noncirrhotic CDI patients reported similar success rates for FMT for recurrent CDI and therapy-resistant severe CDI [51,90,91]. This cohort of patients with LC experienced low rates of severe adverse events related to FMT, including no infections.

TIPS or the transjugular intrahepatic portosystemic shunt has been established as one of the most effective treatments for the complications of portal hypertension. There are several postoperative complications associated with TIPS, such as abdominal hemorrhage, HE, heart failure and nonsurgical infections. An article published in 2021 by Li and collaborators showed the improvement of the liver function in two patients with HE who had undergone TIPS [92]. Table 2 summarizes several key studies from the literature on the impact of Fecal Microbiota Transplantation in Liver Cirrhosis.

The transfer of microbiota could potentially assist patients with hepatic cirrhosis due to viral infections, especially those with hepatitis B virus. Chauhan et al. demonstrated a substantial reduction in the levels of hepatitis B e antigen (HBeAg) after a transfer in the duodenum. More importantly, they observed a decrease in the DNA levels in the studied patients with HBV infection [93].

Beyond this aspect, it is well-known that alcohol has been and remains one of the significant causes of hepatic cirrhosis [94]. However, FMT brings benefits even for these patients, by diminishing the necessity for and usage of alcohol, at least for a short duration [95]. In the long term, a decrease in ascites formation, infections and relapses associated with this vice has been identified [96]. For those with severe alcoholic hepatitis who do not respond to standard steroid treatment, FMT appears to be a promising option and we may see the inclusion of this treatment in future protocols [97].

## 9. FMT in LC and Associated Pathologies

Undoubtedly, hepatic cirrhosis is rarely a standalone pathology, but it coexists with other major diseases. Among these, we can mention diabetes mellitus, hyperlipidemia and arterial hypertension, all of which have correlations with the intestinal microbiota. There are several studies that have investigated how an individual’s pathology affects their microbiome composition [98]. Beyond this aspect, FMT has shown significant benefits in improving metabolic syndrome, which would be an advantage if cirrhotic or hepatic patients were to undergo such treatment [99].

**Figure 2:**
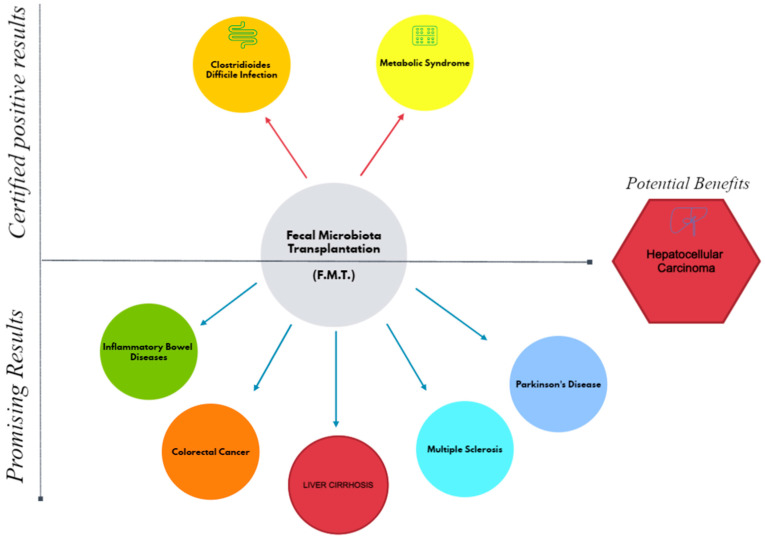
The implications of fecal microbiota transplantation in several major pathologies.

J.S. Bajaj et al. highlightened that LC patients suffering also from CDI were more resistant to treatment and required higher doses, suggesting a strong connection between the liver and the intestine [19]. Furthermore, in associated pathologies, the microbiota suffers additional deficits, making the transfer of microbiota less effective. Encouraging results have also been observed in pathologies such as inflammatory bowel diseases, melanoma, colorectal cancer, Parkinson’s disease, multiple sclerosis and even in the association between CDI and SARS-CoV-2 [100,101,102] (Figure 2).

LC is already known as a proven risk factor for hepatocellular carcinoma; thus, modifying the microbiome could potentially bring benefits either in the treatment or prevention of neoplasms. However, currently, there are no specific cases in the literature where the effects of microbiota transfer have been studied on hepatocellular carcinomas [103].

## 10. FMT in NAFLD/NASH

Hepatic steatosis, a precursor to potential hepatic cirrhosis, is experiencing a rising prevalence [104,105]. This prompts contemplation on whether transplantation could serve not only as a therapeutic measure, but also as a prophylactic strategy, thereby curbing the escalating care costs and enhancing the quality of life for afflicted individuals. Notably, the marked distinctions in dysbiosis among patients with varying stages of non-alcoholic fatty liver disease (NAFLD), non-alcoholic steatohepatitis (NASH), cirrhosis, and hepatocellular carcinoma imply that the restoration of microbial balance could yield substantive benefits in mitigating these pathologies or, at the very least, impeding their progression [106,107].

In 2017, Da Zhou substantiated the effectiveness of fecal microbiota transplantation in mitigating steatohepatitis induced by a high-fat diet, utilizing murine models [108]. Not more than 5 years later, in 2022, Lanfeng Xue et al. conducted a pivotal clinical trial, arguably one of the most impactful in the current literature, underscoring the tangible advantages conferred by fecal microbiota transplantation in individuals diagnosed with NAFLD, compared to those consuming only probiotics [109]. This sophisticated study enrolled 75 participants, of whom 47 underwent microbiota transplantation, providing invaluable insights into the therapeutic landscape of hepatic conditions. Moreover, positive studies on hepatic steatosis could bring additional hope for a successful transplant outcome in patients with hepatic cirrhosis.

## 11. Discussions and Future Perspectives

Despite the growing number of clinical studies on the effects of FMT in hepatic cirrhosis, numerous questions persist without clear answers. The need for a multicenter clinical trial is greater than ever, as it could clarify whether the findings presented in the literature can be generalized and applied to a large scale of patients. Conducting such a study would not only address limitations associated with the route of administration, optimal timing and preparation methods of the transferred material, but also pave the way for the development of an international protocol. This protocol could offer medico–legal coverage for clinicians involved in fecal microbiota transplantation procedures. Uncertainties still exist concerning the patient typology with liver disease that responds effectively to FMT and a study analyzing the response rate of patients based on the Child–Turcotte–Pugh score would be of significant importance. Molecular biology and the way cellular structures interact could bring additional benefits in the near future. Thus, super-resolution fluorescence microscopy and Förster resonance energy transfer (FRET) have the ability to investigate the functionality of biological systems and represent some of the most important tools of the future in medical research [110,111].

Concerns about the safety of the procedure have considerably diminished and it can be confidently stated that FMT is a procedure with satisfactory safety and a low percentage of adverse reactions. Patients are becoming more compliant with the treatment, especially due to the new methods of administration and the significantly high efficacy of FMT in CDI, which has brought optimism both to patients and to individuals who are increasingly attentive and informed about medical advancements.

With the hope that future studies will shed light on the long-term effects of microbiota modification, there is a high probability of discovering whether FMT can be an effective treatment for hepatic failure, leading to increased survival rates and reduced hospitalization periods.

## 12. Conclusions

Fecal microbiota transplantation is likely to become one of the future treatments for hepatic cirrhosis. The existence of concrete studies demonstrating the real benefits of this treatment in hepatic steatosis, viral infections with hepatitis B virus and other significant pathologies could contribute to the prophylaxis of advanced stages of hepatic fibrosis. While literature studies on the positive effects of FMT in established hepatic cirrhosis are not yet entirely convincing and there is a lack of sufficient research on large patient groups to formulate a new treatment protocol, the available data, especially regarding the gut-liver connections, suggest that this pioneering treatment holds promise. In the coming years, it has the potential to revolutionize our approach to cirrhosis treatment.

Within the landscape of advancing medical research, motivating researchers to embark on multicenter clinical trials exploring fecal microbiota transplantation could not only catalyze the development of innovative treatment modalities, but also enhance the depth and significance of specialized literature in the field of hepatic cirrhosis and related conditions.

## Figures and Tables

**Table 1:** Donor Screening.

Initial Screening	Potentially Transmittable Disorder Risk Assessment Questionnaire
Secondary screening	Blood and feces tests
	Hepatitis A (IgM for hepatitis A, total antibodies)
	Hepatitis B (total core antibody, surface antigen and surface antibody for hepatitis B)
	Hepatitis C (HCV antibody test)
	Entamoeba histolytica (test through dipstick and agglutination)
	Treponema pallidum (TPHA)
	HIV (type I and type II)
	Virus Epstein–Barr (viral capsid antigen for IgG and IgM, anti Epstein–Barr virus nuclear antigen)
	Cytomegalovirus (IgG, IgM)
	Human T-lymphotropic virus type I and type II
	Bacteriological and parasitological evaluation (PCR or triple feces test)
	*Clostridioides difficile test (toxin ELISA, PCR or culture)*
	Strongyloides stercoralis (ELISA)
Screening one day prior the donation	In addition to questions regarding stool frequency and pattern, antibiotic use, travel history and recent sexual behavior, the questionnaire also inquires about general health.

**Table 2:** Studies presenting impact of F.M.T. in L.C.

Study	References	Year	Subjects	Findings
M. Grąt et al.	[31]	2015	FMT—40 patients with LC.	The most influencial factor of pre-liver transplantation gut dysbiosis is represented by *Enterococcus*.
Philips et al.	[84]	2017	195 patients with alcoholic liver disease	FMT improved the severity of the disease and survival.
J. S. Bajaj et al.	[19]	2017	20 patients with LC and HE.	FMT in cirrhosis with recurrent H.E led to reduced hospitalizations, improved cognition and reduced dysbiosis.
R. Mehta et al.	[69]	2018	10 patients with recurrent overt hepatic encephalopathy.	60% of patients showed a sustained clinical response at week 20 post-treatment after receiving a single FMT. treatment.
Pringle et al.	[88]	2019	14 patients with CDI and end-stage liver disease.	To cure recurrent CDI in cirrhotic patients, more frozen oral FMT doses are needed.
Olmedo et al.	[17]	2019	4 cases of LC with CDI.	Severe complications in two patients after FMT; cholangitis in the death case was deemed a coincidence. No recurrent CDI episodes in the surviving cases.
Cheng et al.	[89]	2020	63 adults with LC and CDI.	FMT for CDI in LC patients showed low rates of severe adverse events, including no infections.
J. Li. et al.	[92]	2021	2 patients with E.	Improvement in liver function observed in two HE patients who underwent TIPS.

FMT—fecal microbiota transplantation, LC—liver cirrhosis, HE—hepatic encephalopathy, CDI—*Clostridioides difficile* infection, TIPS—transjugular intrahepatic portosystemic shunt.

## Data Availability

Not applicable.
